# Investment case for primary health care in low- and middle-income countries: A case study of Kenya

**DOI:** 10.1371/journal.pone.0283156

**Published:** 2023-03-23

**Authors:** Daniel Mwai, Salim Hussein, Agatha Olago, Maureen Kimani, David Njuguna, Rose Njiraini, Elizabeth Wangia, Easter Olwanda, Lilian Mwaura, Wesley Rotich

**Affiliations:** 1 Economics Department, University of Nairobi, Nairobi, Kenya; 2 Department of Research and Knowledge Management, Futures Health Economics and Metrics Limited, Nairobi, Kenya; 3 Department of Information Technology and Data, Futures Health Economics and Metrics Limited, Nairobi, Kenya; 4 Department of Primary Health Care, Kenya Ministry of Health, Nairobi, Kenya; 5 Department of Community Health, Kenya Ministry of Health, Nairobi, Kenya; 6 Department of Planning and Health Financing, Kenya Ministry of Health, Nairobi, Kenya; 7 Kenya Country Office, United Nations Children’s Fund, Nairobi, Kenya; 8 UHC Secretariat, Kenya Ministry of Health, Nairobi, Kenya; Duke University, UNITED STATES

## Abstract

**Background:**

Primary healthcare (PHC) systems attain improved health outcomes and fairness and are affordable. However, the proportion of PHC spending to Total Current Health Expenditure in Kenya reduced from 63.4% in 2016/17 to 53.9% in 2020/21 while external funding reduced from 28.3% (Ksh 69.4 billion) to 23.9% (Ksh 68.2 billion) over the same period. This reduction in PHC spending negatively affects PHC performance and the overall health system goals.

**Methods:**

We conducted a cost-benefit analysis and computed costs against the economic benefits of a PHC scale-up. Activity-Based Costing (ABC) on the provider perspective was employed to estimate the incremental costs. The OneHealth Tool was used to estimate the health impact of operationalizing PHC over five years. Finally, we quantified Return on Investment (ROI) by estimating monetized DALYs based on a constant value per statistical life year (VSLY) derived from a VSL estimate.

**Results:**

The total projected cost of PHC interventions in the Kenya was Ksh 1.65 trillion (USD 15,581.91 billion). Human resource was the main cost driver accounting for 75% of the total cost. PHC investments avert 64,430,316 Disability Adjusted Life-Years (DALYs) and generate cost savings of Ksh. 21.5 trillion (USD 204.4 Billion) over five years. Shifting services from high-level facilities to PHC facilities generates Ksh 198.2 billion (USD 1.9 billion) and yields a benefit-cost ratio of 16:1 in 5 years. Thus, every $1 invested in PHC interventions saves up to $16 in spending on conditions like stunting, NCDs, anaemia, TB, Malaria, and maternal and child health morbidity.

**Conclusions:**

Evidence of the economic benefits of continued prioritization of funding for PHC can strengthen the advocacy argument for increased domestic and external financing of PHC in Kenya. A well-resourced and functional PHC system translates to substantial health benefits with positive economic benefits. Therefore, governments and stakeholders should increase investments in PHC to accelerate economic growth.

## Introduction

Primary health care (PHC) is essential health care anchored on empirical, scientifically sound, and culturally sensitive approaches and technology. It is accessible everywhere and to everyone in the community through their full involvement, at a reduced and sustainable cost and fosters autonomy and self-determination [1]. The 1978 Alma Ata Declaration shifted focus to health improvement for all from hospital-based care and biomedical innovation and included basic rights, fair treatment and community involvement [2]. The MDGs focussed on treatment and prevention, and unlike the PHC approach, it factored in the social determinants. However, they did not include egalitarianism, equity and public engagement [3]. The 2015 SDGs succeeded the MDGs and provided the impetus to the Alma Ata and Astana principles through other SDGs such as ‘equity’ (SDG 10), ‘community participation’ (SDG 6) and ‘intersectoral collaboration’ (SDG 17 [4].

During the Alma Ata declaration, it was agreed that PHC was to be implemented in eight elements, including education on health problems and how to prevent and control them, development of effective food supply and proper nutrition, maternal and child healthcare, including family planning, adequate and safe water supply and basic sanitation, immunization against major infectious diseases, local endemic diseases control, appropriate treatment of common diseases and injuries and provision of essential basic medication. Kenya added four more elements, including dental health, mental health, HIV/AIDS, primary eye care, Health Management, and information systems [5]. For Kenya to ensure quality PHC for all citizens, and in response to the call by the Astana Declaration 2018, the Ministry of Health developed the Kenya Primary Health Care Strategic Framework 2019–2024. It also aims to scale up establishment of Primary Care Networks [6]. Strengthening PHC should start from the community, dispensaries and health centres, all linking to the hospital to form a network of practice. PCNs link and strengthen health care services by building on a person-centred approach to health which enables proactive, personalized, coordinated and integrated social and health services [7].

Health systems based on high-yielding primary health care can attain better health outcomes fairly and at a reduced cost compared to health systems that overemphasize disease-specific or hospital-based care [8]. Higher investment in PHC is linked to reduced costs, patient fulfilment, reduced hospitalizations and lesser deaths [9]. The Kenyan government made a commitment to achieve universal health coverage (UHC) by the year 2022 and adopted primary health care as the approach to deliver the UHC. This is well articulated in the Kenya Primary care strategic framework 2019–2024 [10]. Universal Health Coverage needs a resilient primary health care as the basis of the health system. However, PHC is yet to deliver on the promises of these declarations [11]. According to the Lancet Global Health Commission on financing primary health care in LMICs remain inadequate is inadequate, access to PHC services is unfair, resource constrained and is dependent on out of pocket expenditure [12]. Consequently, most people avoid primary healthcare facilities to search for specialist care. This stripes PHC of funding, and the resources constraints additionally heighten the issues that drive patients elsewhere [12].

Globally, most countries spend only a third of their health budgets on PHC [13]. Currently, PHC spending ranges between $15 and $60 per capita in LMICs [14]. Increasing primary health care interventions in LMICs could preserve 60 million lives and raise life expectancy to 3.7 years by 2030 [15]. At 80% population coverage, PHC interventions in LMICs cost $350 billion, or approximately $97 per capita [16]. This points to the need of an improved funding to improve PHC delivery and to ensure a responsive health system. An extra investment of roughly US$ 200 to US$ 370 billion annually is needed for an extensive package of health services [15].

Even with incremental gains in health budget allocations after devolution, Kenya still experiences challenges in mobilizing and spending available national resources. The estimation of PHC expenditure is by health care functions. Transfers to universal health coverage programmes, including free primary care services, and transfers to level 5 hospitals constituted 5.9% and 6.7% of the recurrent budget, respectively. Allocations to personnel emoluments decreased from 15.5% in FY 2018/19 to 14.8% in FY 2019/20 before increasing to 17.6% in FY 2020/21. The balance of 7.8% from the 39% that was to be raised from internal revenue (i.e., user fees and sale of medical supplies), was allocated to operations and maintenance [17]. Kenya is consistently below 15% target set by the Abuja declaration for healthcare expenditure forcing people to pay for health care. This regressive form of financing for health means the poorest and most vulnerable bear the greatest burden, pushing many into impoverishment as they pay for healthcare. There is a need to develop a Kenya-specific investment case for PHC and use it as an advocacy tool to increase prioritization and sustainable financing.

There is global evidence that investment in PHC health can earn up to ten times in returns, creating the need for a country-specific study [18]. However, the scope of PHC services, the amount invested in PHC, the additional amount that should be invested in PHC, and how to address the existing funding gap remain to be determined. Moreover, there is little guidance on the efficient use of health resources for improved development and performance of their PHC systems to make the most of the health gains of the population. Returns on investments generated by investing in PHC remain unclear at National and County government levels. As such, an analysis on the investment case in PHC including the PHC networks will improve health and support a productive society. This study aims to develop a Kenya-specific investment case for PHC by estimating the return on investments and describing the impact of scaling up PHC interventions.

## Methodology

This study used a cost-benefit approach in which the cost of PHC delivery was computed against the economic benefits scale-up action.

### Cost of the PHC priority areas

To estimate the costs of the PHC interventions, we employed the Activity-Based Costing (ABC) approach due to its usefulness in priority setting and linking output to cost. The costing focussed on the provider perspective and primary data was collected in the following areas: stunting, non-communicable diseases, anaemia, Tuberculosis, Malaria, HIV and other diseases/conditions. We estimated the implementation costs the Kenya Primary Healthcare Strategic Framework (2019–2024) including the cost of establishing and maintaining functional Primary Healthcare Networks.

Costing data was obtained from various sources. The activities to be costed were extracted from the PHC strategic framework 2018/19–2023/24. We conducted consultative meetings to refine the activities and establish the implementation path for each activity prioritized in the strategic framework. The Ministry of Health provided the data on input prices and the frequencies of the activities while the thematic teams discussed the optimal inputs needed to implement each of the activities in each of the strategic areas of the framework. These thematic groups included Leadership and Governance; Human Resource for Health; Service Delivery; Health Financing; Commodity supply and infrastructure; and Health information, technology, and innovations. Besides, data sources for the activities needed for effective operationalization of the PCN varied as well. A PCN is an administrative health region comprising a primary healthcare referral facility (hub) and several other primary healthcare facilities (spokes). PCNs improve access to primary health care services for patients and coordinate with other hospitals to improve the overall operational efficiency of the network. The PCNs are designed to have a modified ‘hub and spoke’ model. The modified hub and spoke emphasizes the population’s needs with the community as the entry-level to the health system. The hub should be a level 4 facility (sub-county, faith-based or private hospital). It will support the spokes, comprising levels 2 and 3 facilities and level 1 community health units (CHUs). While in some regions, a sub-county may constitute one PCN, some sub-counties, especially geographically vast counties, may have more than one hub, hence more PCNs. In such situations, an SCHMT shall have oversight over many PCNs [19].

Cost data for human resource required for a PCN was extracted from the Ministry of health staffing Norms and standards as stipulated in the Ministry of Health, Human Resources for Health Norms and Standards Guidelines for The Health Sector, 2014–2018 [20]. The guidelines outline the needed investments for fair and satisfactory capacity to deliver the Kenya Essential Package for Health. Data on commodities and supplies was based on projections and quantification and Health Products and Technologies Supply Chain Strategy 2020–2025. The Kenya Harmonized Health Facility Assessment (KHFA) provided information on past investment and the availability of each of the PCN investment areas. We employed an incremental costing approach to estimate the extra investment required for the PHC for 5 years, with specific costing of the cost of setting up and operationalize a PCN in Kenya. The costing for PCNs included these four scenarios: the total cost of setting up a PCN afresh, assuming no prior investments have been made to the PCN; Cost of PCN based on existing Need/Gaps; Cost of PCN with existing HRH and infrastructure; and Cost of PCNs with existing HRH, Commodities/Supplies, and Infrastructure. The first scenario estimated the total cost of setting up a PCN assuming zero investments. The cost estimates included the cost of constructing three health centres and a level four facility, the cost of investing in supplies, assuming that there was none in the system and the cost of deploying new human resources. The second scenario estimated the cost of setting up a PCN based on need. This need was established based on evidence from health facility assessments and the incremental cost estimated. In the third scenario, we assumed that the country is constrained, triggering the need to rationalise human resources and use the current infrastructure. Therefore, the costing should have included the infrastructure and human resources. The fourth scenario was costed based on the assumption that the PCNs used the existing infrastructure, human resources and supplies. Noteworthy is the fact that there exist challenges with human resources and commodities.

An excel-based data tool was used to capture specific inputs needed for each investment area, including the data from past health system and facility assessments. The tool was also customized to capture country-specific data to enable counties to estimate the total resources needed for operationalizing PCNs in their county. The current prices were adjusted for medical and overall inflation depending on the input type. Since the costing covered one year, it was expected that the bulk of the inputs would not change prices during the period. Data analysis was conducted using an Excel-based tool that had the provision for activity/sub-activity, costing assumptions, inputs specification, input price, frequency of occurrence, recurrence of the activity, and total cost of the activity. See the supplementary material on PHC costing and PCN assumptions.

### Impact analysis

The One Health Model was used to estimate the health impact of operationalizing PHC based on the indicators provided [21]. The model enables joint planning, costing, budgeting, impact and financial space analysis and combines disease programmes and health systems. It has nine inbuilt modules used to estimate the impact of several variables such as years of life saved, and deaths averted after scaling up interventions. In this analysis, we used LiST, NCD, AIM, Malaria, TB, and WASH modules to estimate the health impact of scaling up PHC interventions as per the PHC Monitoring and evaluation framework. We also used it to estimate the cost-saving by shifting the services delivery from high-level facilities to PHC facilities. Within the one health model, the country target was to shift 70% of healthcare services from level four going down. In the status quo, 70% of services are delivered in levels four, five and six. With the PHC targets, 70% of PHC services should be offered in levels two, three and four. The model was set at status quo, and cost of delivering the services was estimated. Similarly, estimates of shifting the 70% services to levels two, three and four were calculated in line with the country’s target. The difference gives us the cost savings.

To estimate the number of lives saved following the scale up of PHC interventions, we used the Lives Saved Tool (LiST), which projects future deaths by year based on certain user-provided inputs, including the coverage rates of health interventions. Two LiST projections including a “baseline” projection, which projected the number of deaths with no changes to current coverage rates or any pre-loaded inputs were run. Besides, we ran an “intervention” projection, which forecast deaths over the same period, but assumed increased coverage rates for PHC interventions. The same analysis was done for NCD, AIM, Malaria, TB, and WASH modules. In line with the country’s goal of treating diseases in primary healthcare facilities, the scale of PHC was projected at 40%-70%.

The scale-up of these interventions was assumed to take place evenly over 5 years, after which no further increase in coverage was assumed. This was partly informed by the 5-year planning cycle in Kenya and in line with the PHC framework. The number of lives saved each year was calculated as the difference between the baseline and intervention projections. Finally, we quantified the ROI by estimating monetized DALYs based on a constant value per statistical life year (VSLY) derived from a VSL estimate. There is uncertainty in VSL and it does not represent the inherent value of life but condenses actual and stated trade-offs people make in choosing between money and small changes in mortal risk [22]. The VSLY is provided by the residue of life expectancy and age of death. We used the Value of Statistical Life-Year for Kenya which is Ksh. 358,567 [23]. Costs are presented in 2022 US dollars, the year of the costing study Costs and benefits were discounted at an annual rate of 3%, consistent with economic guidelines [24].

## Results

### Costs

The total projected cost for implementing all the activities prioritized in the Kenya PHC strategic framework from 2019 to 2024 is Ksh 1.65 trillion (USD 15,581.91 Billion). Human Resource for Health is the main cost driver accounting for 75% of the total cost, followed by commodity supply and infrastructure, which accounts for 13% of total costs. Service delivery accounts for 12% of total costs. Table 1 shows the estimated cost of priorities activities for each of the strategic areas in the Kenya PHC strategic framework.

**Table 1:** Projected resource need for PHC framework by strategic areas (Ksh. Billion).

Strategic Direction	2019/20	2020/21	2021/22	2022/23	2023/24	Total	Proportion
Strategic Direction 1: Leadership and Governance	0.69	0.18	0.19	0.20	0.21	1.46	0%
Strategic Direction 2: Human Resource for Health	215.38	230.30	246.39	263.60	282.02	1,237.69	75%
Strategic Direction 3: Service Delivery	110.95	21.74	21.70	21.30	22.47	198.16	12%
Strategic Direction 4: Health Financing	0.42	0.24	0.25	0.26	0.27	1.45	0%
Strategic direction 5: Commodity supply and infrastructure	38.51	40.68	42.53	44.40	46.46	212.58	13%
Strategic Direction 6: Health information, technology, and innovations	0.10	0.06	0.06	0.06	0.07	0.34	0%
**Grand Total (KES)**	**366.06**	**293.19**	**311.12**	**329.82**	**351.49**	**1,651.68**	**100%**
**Grand Total (USD)**	**3.33**	**2.67**	**2.83**	**3.00**	**3.20**	**15.02**	

Table 2 reports the costs of setting up and operationalizing a PCN in Kenya under four assumptions. The total cost of setting up a PCN afresh is the highest at Ksh. 4,875.77 million (USD 44.33 million) compared to the cost of setting a PCN using the existing HRH, commodities/supplies and infrastructure at Ksh. 99.02 million (USD 0.90 Million). See Table 2.

**Table 2:** Cost of setting up Primary Health Care Networks (PCNs) under various assumptions.

Cost Items	A PCN afresh	Based on existing Need/Gaps	Under existing HRH and infrastructure.	Under existing Commodities / Supplies, and Infrastructure.
(i)Health service delivery.	16.31	16.31	16.31	16.31
(i)Human resources for health	3,010.61	411.48	52.36	52.36
(ii) Infrastructure, equipment, water, sanitation, electricity, and other capital costs.	1,279.45	78.66	78.66	-
(ii)Supplies and commodities	503.36	178.11	178.11	-
(iv) Annual planning and forecasting–appropriate mechanism for planning and budgeting.	13.02	13.02	13.02	13.02
(vi) Communication and information.	0.62	0.62	0.62	0.62
Capacity building.	2.69	2.69	2.69	2.69
Operations and maintenance.	49.16	13.47	13.47	13.47
Operations and management	0.56	0.56	0.56	0.56
**KES (Millions)**	**4,875.77**	**714.91**	**355.79**	**99.02**
**USD (Millions)**	**44.33**	**6.50**	**3.23**	**0.90**

### Health impacts

The total short-term benefits that can accrue to the nation from the operationalization of the primary health care interventions are assumed to accrue over the next five years. The PHC interventions are projected to have a significant impact. The health impact is the same for all 4 scenarios reported in Table 2 above. The estimates of the annual benefits saved from the diseases for the next five years are shown in Table 3.

**Table 3:** Health impact areas of PHC intervention.

Health impact	Kenya: 2020–2024
Number of child deaths averted	25,413
Number of maternal deaths averted	6,658
Number of still birth averted	15,009
Number of cases of stunting averted	410,307
Number of cases of anaemia in pregnant women averted	251,926
Number of malaria deaths averted	20,229
Number of cases of malaria averted	778,069

The sum of these health impacts results in a potential 64,430,316 DALYs averted. The YLL and YLD are shown in Table 4.

**Table 4:** Health impact of PHC interventions on Disability Adjusted Life Years (DALYs).

	Kenya—2020–2025		
Measure of Burden of disease	YLL	YLD	Total
YLL averted	1,951,010	2,587,432	4,538,442
YLD due to stunting	23,765	52,930	76,695
YLD due to NCDs	1,248,432	3,627,236	4,875,668
YLD due to anaemia	5,432	13,100	18,532
YLD due to TB	1,143,432	1,721,200	2,864,632
YLD due to Malaria	2,247,878	1,366,778	3,614,656
YLD due to HIV	4,686,687	8,567,534	13,254,221
YLD due other diseases/conditions	13,417,427	21,770,043	35,187,470
Total DALYs	24,724,063	39,706,253	64,430,316

The financial implication of not investing in PHC is Ksh 21.5 trillion (USD 204.4 Billion) over five years. The benefits resulting from shifting services from high-level facilities (Level 5 & 6) to PHC facilities were estimated as health system savings, amounting to Ksh. 198.2 billion (USD 1.9 Billion) over five years. These cost savings can fund the scale-up of PHC in the short term and thus reduce the funding gap associated with scaling up PHC in Kenya.

### Return on investment (ROI)

The ROI was established by comparing the benefits versus the costs. The cost of investing in PHC interventions is Ksh. 25,635 (US $ 246.5) per DALY averted. Using the Value of Statistical Life-Year for Kenya that is Ksh. 358,567 (US $ 3,448), it is also estimated to yield a benefit-cost ratio of 16:1 in 7 years, which is indicative of excellent value for money from this investment.

## Discussion

This study examined the benefits and associated costs of investing in PHC interventions in Kenya. Our analysis suggests that investing in PHC interventions would generate health system savings and gains in the wider health and social care economy. Every $1 invested in PHC interventions saves up to $16 in spending on conditions like stunting, NCDs, anaemia, TB, Malaria, maternal and child health morbidity. This finding is consistent with that of a systematic review which concluded that investing in local public health interventions are cost-saving, and offer substantial ROI [25].

A recent study reported that the ROI of scaling up community health interventions in Kenya generated up to 9.4 times return on investment [26]. Another analysis estimated the yearly economic gains of a community health workers (CHWs) system across sub-Saharan Africa would report a positive return as high as 10:1 against an annual system cost of $2.2 billion. Also, approximately $750 million in economic losses could be averted annually via CHW scale-up [27]. While the demonstrated ROI for PHC is high, there are challenges with comparing the published ROIs across different interventions due to varying assumptions of program costs and benefits, and the possibility of including or excluding beneficiaries to whom a return flow. The generalisability of the interventions will vary thus the need to have a standardized way of describing the scope of PHC. This analysis suggests that investments in PHC are sustainable and can result in long-term economic payback and growth.

The study findings suggest that it is costly to start investing a fresh in PCNs. In contrast, the incremental cost of setting up a PCN based on the need established during health facility assessments offer cost savings of 37.83 million USD. This can be attributed to the fact that starting a fresh requires an initial capital investment on HRH. Moreover, investments in recurring resources to cover the costs of the remuneration of additional health workers, equipment, supplies and consumables are needed. Consequently, governments and international donors must harmonize their support interventions with the PHC vision and objectives. Key to the process of such implementation is to keep an integral overview of PHC systems, in order to ensure the compatibility and complementarity of the PHC components. Additionally, political commitment is the foundation of the successful investment in PHC because the long-term PHC reform goes beyond the length of a typical political cycle.

Maximizing the economic benefits of PHC is also dependent on high yield interventions that ensure better health outcomes. Our analysis shows a high return on investment for some interventions within PHC including malaria, stunting and anaemia. There are currently no recommendations on the specific mixes of PHC interventions. This points to the need to assess the epidemiological and economic efficiency of the different PHC intervention mixes to recommend optimal strategies and estimate further cost savings accrued through technical and programmatic efficiencies. This evidence can direct policymakers to on areas of PHC to prioritize on after assessing barriers and facilitators PHC implementation. It will also emphasizes explicit consideration of costs and consequences, and provide critical information on efficient resource use [28].

This study had three limitations. First, we assume a limited time horizon for the PHC interventions while most interventions modelled have lifetime benefits. Second, we assume patients seeking care at PHC facilities do not get additional interventions during the time horizon stated. Third, this analysis only reports service delivery and alignment toward primary health care. More evidence on the economic impact of multisectoral policies and determinants of health and empowerment of individuals, families and communities on PHC is required.

This is the first modelled analysis of the return on investments associated with PHC in Kenya. The projected Ksh 1.65 trillion is way above the FY 2020/21 allocation. The MOH allocated Ksh 3.8 billion, equivalent to 5.9% of the MOH recurrent budget allocation. This allocation was earmarked for universal health coverage, comprising Ksh 2.9 billion to grants for scale-up of universal health coverage and a further Ksh 900 million to the free primary healthcare programme. This amount was a decrease from the previous years’ allocation for universal health coverage of Ksh 11.0 billion in FY 2018/19 and Ksh 6.5 billion in FY 2019/20 [17]. However, the positive and strong return on investment suggested here is a strong justification for National and County Governments in Kenya as well as donors and other stakeholders working in the healthcare sector to increase investments towards PHC in Kenya. High-level advocacy to policymakers and donors is needed to ensure sustained financing for PHC. They also need some political will for implementation. There should be a rerouting of household funding to a prepayment mechanism to reduce the incidence of catastrophic health expenditure. Collaboration between the national level and the county level in operationalizing the PHC approach should also be enhanced to explore alternative funding sources to meet the cost of PHC scale up in the short term. Additionally, referral systems from PHC to higher levels of care is crucial area of investment that may be more impactful.

## Supporting information

S1 File PCN key assumptions. (DOCX)

S2 File PHC strategic plan costing tool. (XLSX)
