# Supplementary material for: Investment case for primary health care in low- and middle-income countries: A case study of Kenya
Source: PLoS One. 2023 Mar 23;18(3):e0283156. doi: 10.1371/journal.pone.0283156 (PMC10035909; doi:10.1371/journal.pone.0283156)
Supplement: S1 File — (DOCX) [file pone.0283156.s001.docx]

**Primary Care Network (PCN) Cost Assumptions**

| **Key Areas** | **Key Assumption** | **Source/Reference** |
| --- | --- | --- |
| **Staff** | Staff requirement per level of facility was based on Norms and availability as per the KHFA report | KHFA 2018-2019 |
|  | Salary per carder based on SRC salary Scales | SRS salary scales |
| **Capacity Building** | Based on costed list of activities, where key inputs are quantified and costed based on frequencies | Activity Based Costing |
| **Infrastructure, Equipment, water, sanitation, electricity, and other capital cost** | Infrastructure, Equipment, water, sanitation, electricity, and other capital cost requirement per level of facility was based on Norms and availability as per the KHFA report | KHFA 2018-2019. Annex Table 10 |
|  | Based on government costing on construction and rehabilitation of structures, categorized as large scale, medium and small-scale upgrade |  |
|  | Where availability is <50%-Large scale upgrade is required, 50-75%-Medium scale upgrade is required, >75%-Small scale upgrade is required. |  |
|  | Equipment; Based on government costing for equipment requirement per level of facility |  |
|  | Equipment; Based on government costing for furniture requirement per level of facility |  |
| **Operation and management** | Based on costed list of activities, where key inputs were quantified and costed based on frequencies |  |
| **Operation and maintenance** | Operation and maintenance requirement per level of facility was based on Norms and availability as per the KHFA report | KHFA 2018-2019. Annex table 10 |
| **Supplies and Commodities** | Supplies and Commodities requirement per level of facility was based on Norms and availability as per the KHFA report | KHFA 2018-2019. Annex table 15 |
|  | Mean cost of Drugs and supplies per level of facility was factored in | MSH PHC costing, Table 7 |
| **Annual planning and forecasting-approriate mechanism for planning and budgeting** | Based on costed list of activities, where key inputs are quantified and costed based on frequencies | Activity Based Costing |
| **Communication and information** | Based on costed list of activities, where key inputs are quantified and costed based on frequencies | Activity Based Costing |
| **Health service delivery** | Based on costed list of activities, where key inputs are quantified and costed based on frequencies | Activity Based Costing |
